# Fixation free laparoscopic obliteration of inguinal hernia defects with the 3D dynamic responsive scaffold ProFlor

**DOI:** 10.1038/s41598-022-23128-6

**Published:** 2022-11-08

**Authors:** Giuseppe Amato, Antonino Agrusa, Pietro Giorgio Calò, Giuseppe Di Buono, Salvatore Buscemi, Adriana Cordova, Guido Zanghì, Giorgio Romano

**Affiliations:** 1 Department of Surgical, Oncological and Oral Sciences, University of Palermo, Via L. Giuffrè, 5, 90127 Palermo, Italy; 2 Department of Surgical Sciences, University of Cagliari, Cittadella Universitaria 09042 - Monserrato (CA) - ITALY, Italy; 3 Department of General Surgery, University of Catania, Via Santa Sofia, 76 - 95123 Catania, Italy

**Keywords:** Clinical trials, Therapeutics

## Abstract

Laparoscopic TAPP/TEP approaches are well-established options for the cure of inguinal hernias. As in the open approach, mesh fixation and poor-quality biologic response represent controversial questions and are a source of concerns. Furthermore, hernia defect patency represents another problem which seems not well acknowledged among surgeons. These problems are considered the cause of frequent intra and postoperative complications. To overcome these concerns, recently a different concept of cure has emerged. Based on a newly developed dynamic responsive 3D scaffold named ProFlor, a permanent hernia defect obliteration has been finalized. Following its inherent centrifugal expansion due to its dynamic responsivity, this hernia device is positioned fixation free within the defect and induces a probiotic biological response allowing for the re-establishment of the degenerated inguinal barrier. A laparoscopic approach with the 3D scaffold has been tested on 71 patients to demonstrate its effectiveness in reducing intra and postoperative complications. The operated patients presented with bilateral and/or recurrent inguinal hernia. Overall, 122 hernia defects were obliterated with 119 dynamic responsive scaffolds. The procedures were carried out from January 2018 to January 2022 with a defined protocol and detailed procedural steps. The laparoscopic technique with the 3D hernia scaffold allowed for fixation free placement, permanent defect obliteration and dynamically induced regenerative effects. The technique proved effective in reducing intra and postoperative complications. In particular, early postoperative pain and discomfort significantly decreased. No chronic pain and no recurrences were reported during follow up. The results achieved with the described laparoscopic technique seem to embody an innovative concept for inguinal hernia repair. Fixation free, dynamic responsive, permanent defect obliteration, histologically proven regenerative effects are the distinctive features of this 3D scaffold. It seems to embody a more physiological and pathogenetically coherent concept of cure, thus improving treatment results of this widespread disease.

## Introduction

Many techniques and several types of implants are used for the repair of inguinal hernias. The concept of reinforcing the herniated groin by deploying a flat mesh, usually made of polypropylene, has demonstrated effectiveness in reducing the high recurrence rate of the pre-prosthetic era^1^. Nowadays, all implants used for hernia repair are flat, static, passive and do not cope with movements of the abdominal wall. Open anterior or posterior repair, laparoscopic transabdominal preperitoneal (TAPP) or totally extra-peritoneal (TEP) are two of the most popular herniorrhaphy methods, nevertheless the surgical community is still searching for a gold standard in the treatment of this disease^2,3^. In the past two decades, laparoscopic hernia repair has been more frequently reported as the method of choice, especially in the case of recurrence after open anterior repair or bilateral inguinal hernias^4^. However, laparoscopic TAPP and TEP present several challenges related to anatomical conditions, surgical technique and prosthetics^5^. For large defects, to avoid mesh invagination through the hernia opening under the push of the abdominal viscera, larger and heavier meshes with multiple fixation tools are used. This implies wider peritoneal dissection and increased risk of intra- and postoperative complications^5,6^. A consequence of broader tissue dissection and use of wider/harder meshes is an increase of fibrotic bridles and implant induced scar plaque in the delicate inguinal area that potentially increases postoperative pain and is considered harbinger of chronic pain syndrome^7–15^. Furthermore, the patency of the hernia defect after flat mesh deployment embodies another challenge whose relevance has not been adequately considered nor addressed. Leaving the inguinal hernia defect permanently open after mesh deployment is considered the most common cause of recurrence after laparoscopic repair^16^. To resolve these problems, a novel concept has recently been developed. It concerns the use of a 3D dynamic responsive scaffold that is introduced into the hernia opening achieving a permanent defect obliteration^17–19^. This 3D hernia device is made of polypropylene material like conventional hernia prosthetics but owns inherent springiness, is positioned fixation free and, moving together with the groin, induces a probiotic biologic response. Modulated by specific growth factors, the development of newly formed connective tissue, vessels, nerves and muscles within the 3D scaffold has been documented in a series of scientific studies^19–24^. These proprietary regenerative effects, likely result of its intrinsic dynamic responsivity, allow the categorization of this new inguinal hernia device not as a conventional implant but rather a regenerative scaffold. Already used in inguinal hernia repair with open approach, recently a specific laparoscopic TAPP approach has been finalized aimed to further reduce surgical trauma achieving permanent inguinal defect obliteration^25–27^. The present report highlights the outcomes of this novel laparoscopic technique with ProFlor in a cohort of patients suffering from inguinal hernia.

## Materials and methods

### Compliance with ethical standards

The research has been approved by the Ethics Commission of Policlinic Hospital of the University of Palermo, Italy—Approval ID: 10—November 11, 2020.

### Human and animal right

The investigation was carried out in accordance with the Declaration of Helsinki for experiments involving humans. Informed consent was obtained for experimentation with human subjects.

### Registration

ClinicalTrials.gov—Registration ID: NCT04718298—Registration date: 22/01/2021. https://clinicaltrials.gov/ct2/show/NCT04718298?term=Proflor&draw=2&rank=2.

### Participants and tools

**Figure 1:**
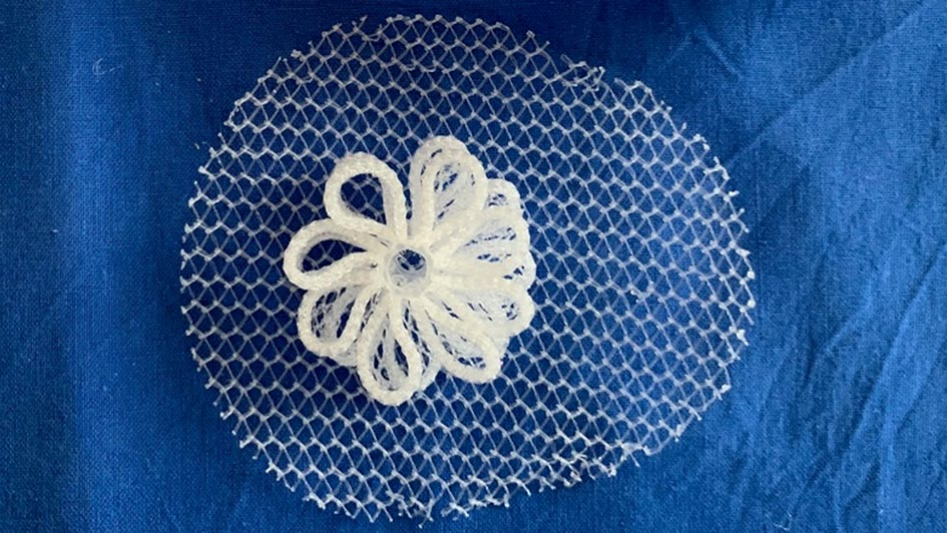
Regenerative scaffold ProFlor E with 40 mm sized multilamellar 3D core and 8 × 10 cm oval flat mesh connected at the center of the posterior surface.

From January 2018 to January 2022, 71 individuals underwent laparoscopic hernia defect obliteration using the 3D dynamic responsive scaffold ProFlor type E. This 3D hernia device is made from low weight, large porous polypropylene, has a multilamellar cylindrical 3D structure with reinforced edges, and is available in two sizes: 25 and 40 mm in diameter, both types 15 mm in thickness. The center of the scaffold core is connected on one surface to an oval shaped flat mesh measuring 8 × 10 cm, which is intended to be deployed to cover the inguinal and femoral area counterfacing the peritoneal sheath (Fig. 1). The 3D core of the scaffold is compressible on both longitudinal and transversal planes. Endowed with inherent dynamic responsivity, it contracts and relaxes with the movements of the groin. Its proprietary centrifugal expansion allows fixation free positioning in the hernia defect for permanent obliteration.

Inclusion criteria for patients of the cohort undergoing inguinal hernia repair were:

- Unilateral recurrent inguinal hernia previously operated with anterior open approach.
- Bilateral primary inguinal hernia or recurrent unilateral + primary hernia on the contralateral groin.
- Hernia defect diameter < 37 mm preoperatively assessed by US/CT scan.
- Age between 18 and 85 years.
- ASA score < IV.
- BMI < 34.

Details of inclusion criteria and demographic data of the patients are included in Table 1. All patients of the cohort were male. Mean age was 54 (range 33–78) and mean BMI 27.8 (range 25–31).

**Table 1:** Inclusion criteria and patient demographics.

Inclusion criteria		
Unilateral recurrent inguinal hernia previously operated with open approach		
Bilateral primary inguinal hernia or one unilateral recurrent hernia and one primary hernia on the contralateral groin		
Hernia defect diameter < 37 mm preoperatively assessed by US/CT scan		
Age between 18 and 85 years		
Asa score < IV		
BMI < 34		

Patients enrolled		
Total		71
Gender	Male/Female	71/0

Demographics		
Age (mean)	54 years (range 33–78)	
BMI (mean)	27.8 (range 25–31)	

Informed consent was obtained from all the participants involved in the experiment.

### Preoperative clinical details

**Figure 2:**
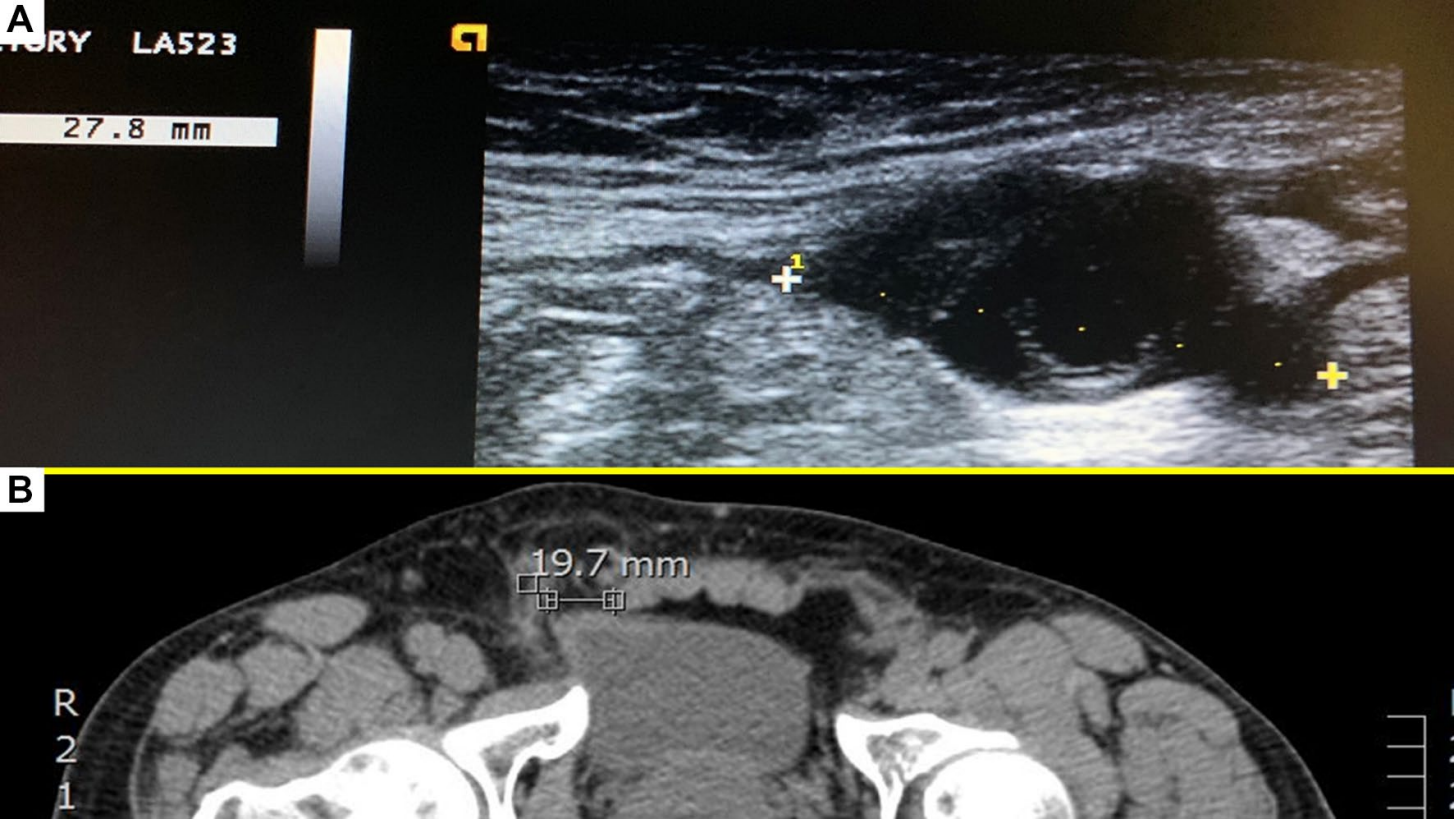
Preoperative hernia defect assessment. (**A**) Ultrasound image of midsized defect of the inguinal backwall. (**B**) CT scan showing small inguinal hernia defect in the right groin.

Preoperative defect measurement carried out with US and/or CT scans was considered mandatory for all patients (Fig. 2A,B). The aim of assessment of the hernia defect is to preoperatively determine the size of the implant core to be chosen for the procedure, e.g. a hernia opening > 37 mm was not contemplated and considered an exclusion criterion. Twenty-seven patients presented with bilateral inguinal hernia and among these 12 had double ipsilateral hernias. Eighteen patients suffered from recurrent inguinal hernia after anterior open approach. Three inguinal hernia patients, one of whom was operated for recurrence, had concomitant femoral hernia.

### Surgical procedure

**Figure 3:**
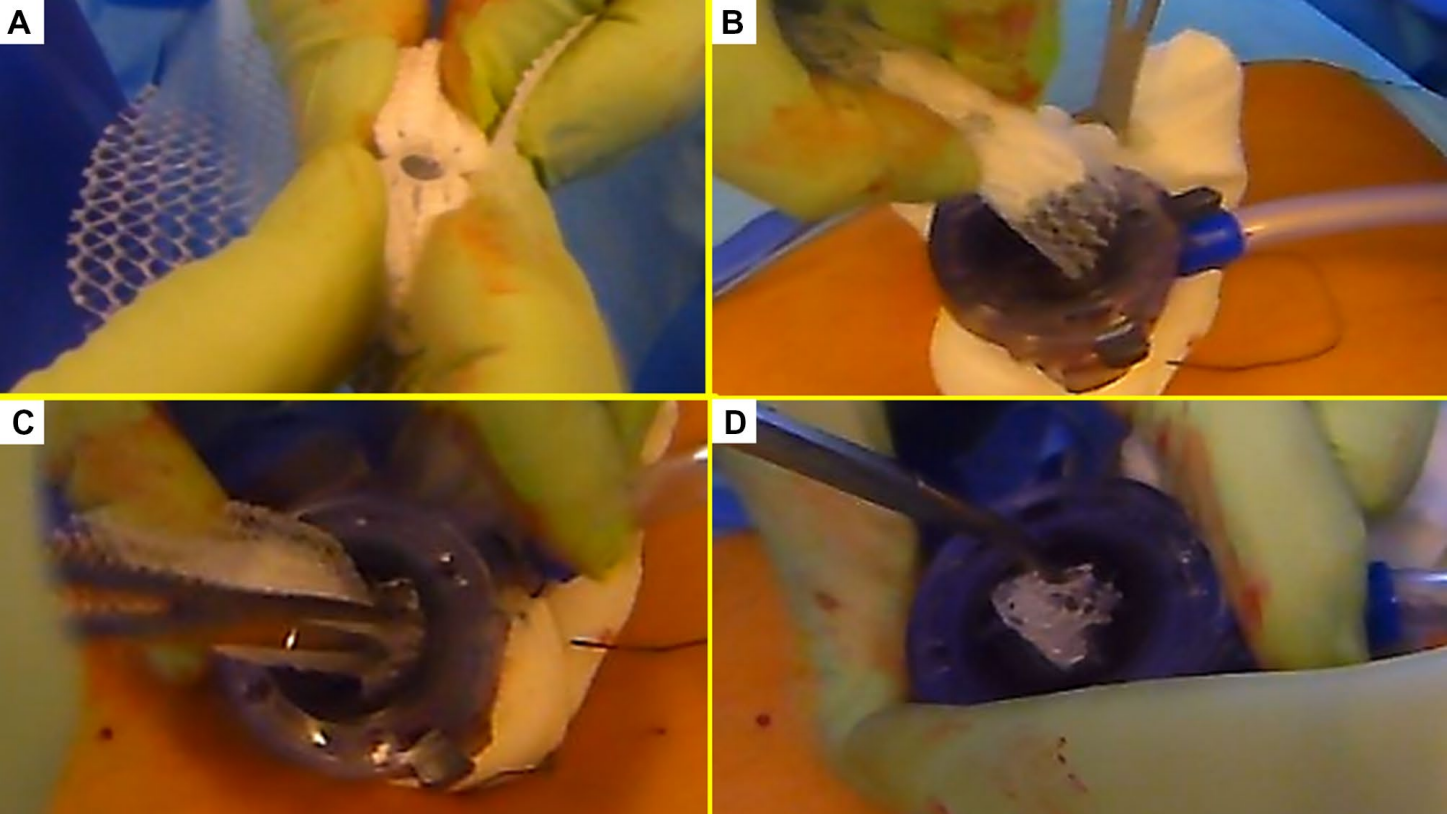
Procedural steps for delivery of ProFlor E into the abdominal cavity. (**A**): 3D core of ProFlor E is squeezed along its longitudinal axis. (**B**) 3D core of ProFlor E is wrapped over the connected flat part before introduction into a 12 mm trocar. (**C**) Forceps guided funneling of ProFlor E through the trocar channel. (**D**) 3D core of ProFlor E has already passed through the 12 mm trocar channel.

**Figure 4:**
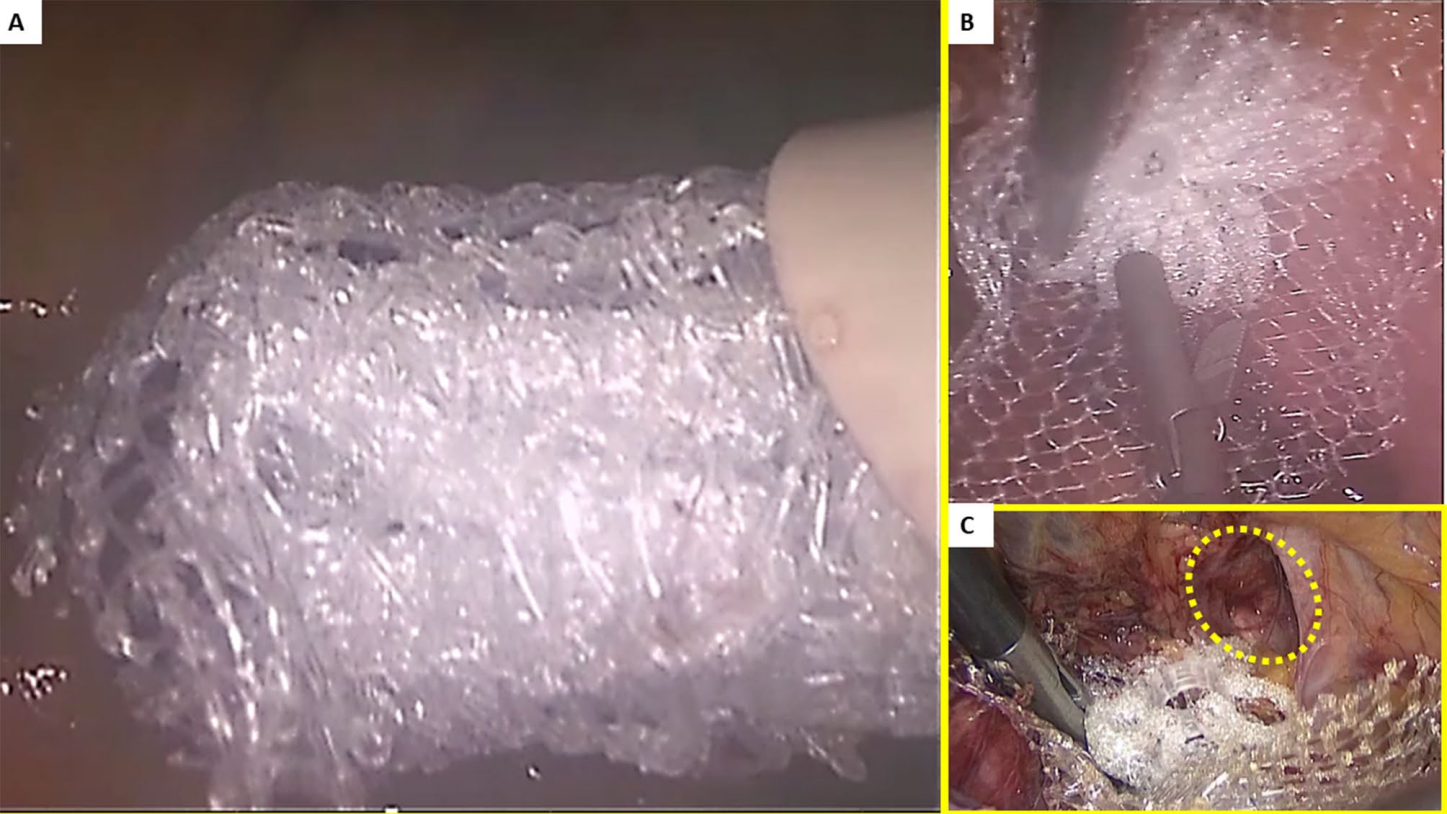
(**A**) The compressed 40 mm sized ProFlor E scaffold is funneled across the 12 mm large trocar channel into the abdominal cavity. (**B**) Once delivered the 3D scaffold promptly regains the original shape. (**C**) A 25 mm ProFlor E is approached to the hernial opening ready to be positioned within the defect (yellow circle).

**Figure 5:**
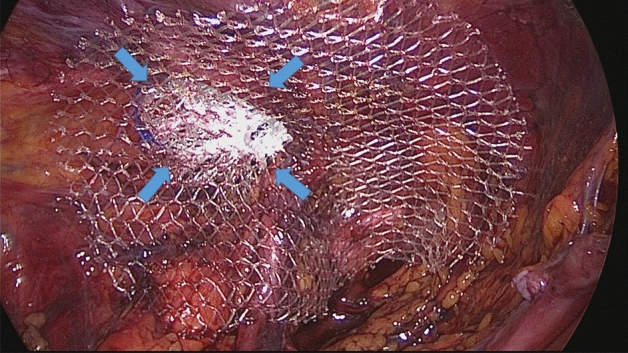
A 40 mm Proflor E, positioned in fixation free fashion, squeezed within the left sized indirect hernia opening achieves a complete defect obliteration. The flat part of the hernia device completely covers the medial and supravesical inguinal fossae as well as the femoral area.

**Figure 6:**
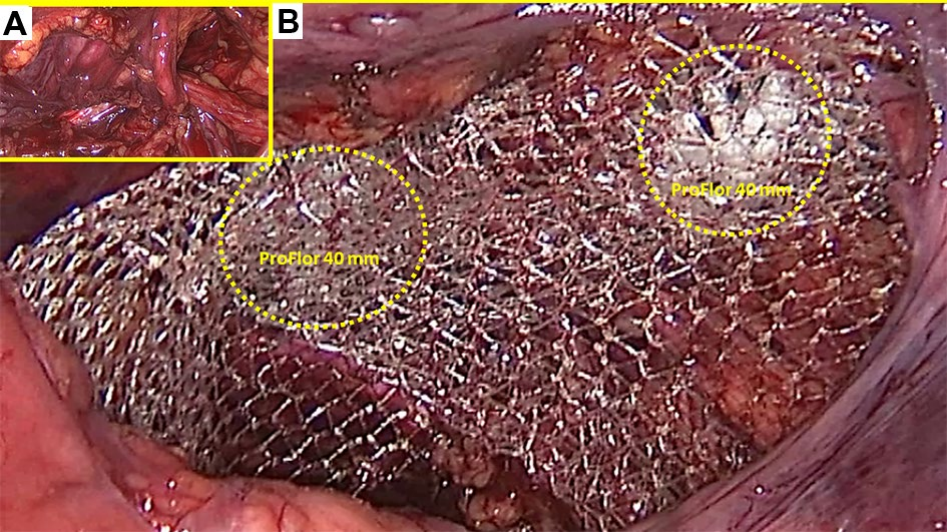
(**A**) preperitoneal aspect of a right sided groin showing two large ipsilateral hernias composed by one direct the medial inguinal fossa and one indirect in the lateral fossa. (**B**) a couple of 40 mm ProFlor E have been already delivered to fixation free obliterate the two openings of the ipsilateral hernias. The flat parts of both 3D scaffolds completely cover the inguinal and femoral area.

All surgeries were carried out in general anesthesia with TAPP technique. A protocol-based antibiotic prophylaxis with a single dose of a cephalosporin was administered one hour before operation. After establishing pneumoperitoneum, three trocars were placed at the umbilical level (12 mm large optic in the navel and two working ports over the right and left rectus muscle); all working ports were inserted under direct vision. Once the hernial defect was identified and the visceral protrusion pulled back to the peritoneal cavity, a dissection of parietal peritoneum to expose the inguinal backwall was made. Adhesiolysis of hernia sac followed. The dissected hernia sac was then returned to the abdominal cavity. After reducing the hernia, the ProFlor implant, rolled upon the flat part and compressed along the longitudinal axis, was introduced through the 12 mm trocar (Figs. 3A–D, 4A). Once delivered into the abdominal cavity, the springiness of the 3D scaffold allows a prompt regain of the original shape (Fig. 4B). Then, with simple maneuvers, the core of ProFlor E was inserted and positioned in the hernial defect with the edges facing the borders to fully obliterate the hernia opening (Figs. 4C, 5). Once the hernia device was firmly delivered into the defect, a simple test carried out by a repeated tentative of pulling out the flat part of the scaffold confirmed the permanence of the 3D core of the scaffold within the defect. Thanks to the centrifugal expansion of the multilamellar 3D core, no sutures or other fixation methods were needed to hold the ProFlor E in place, in any of the patients. At this stage, the flat mesh of the implant could be deployed to cover the inguinal and femoral area (Figs. 5, 6). Closure of the peritoneal flap finalized the procedure.

### Follow up protocol

Patients were discharged within 24 h of surgery with a prescription for an analgesic prophylaxis based on oral administration of 1000 mg paracetamol 3 times per day until the third postoperative day. Follow up was carried out starting after 1 week, 4 weeks, 3 months, 6 months and each following year postoperatively. The protocol included the use of the visual analogue scale (VAS) to assess pain, and US control of the operated groin to demonstrate the permanence of the 3D scaffold in the defect. To highlight the quality of tissue incorporation within the 3D hernia device through the assessment of signal intensity, an MRI of the lower pelvis was programmed between 6 and 12 months, post-operation.

## Results

**Figure 7:**
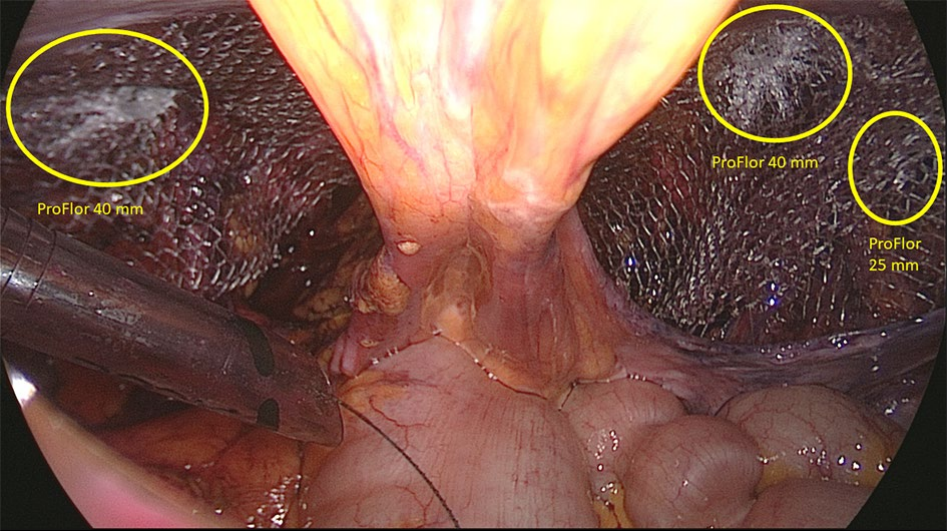
Panoramic view of the lower posterior abdominal wall dissected for TAPP repair of bilateral groin hernias. On the left side an indirect hernia has been obliterated with a 40 mm ProFlor. On the contralateral groin a concomitant double ipsilateral hernia defect (one in the medial inguinal fossa and one in the lateral fossa) has been managed by delivering one 40 mm ProFlor in the direct defect and one 25 mm in the indirect defect. The flat parts of all three devices completely cover the inguinal and femoral areas of both groins.

**Figure 8:**
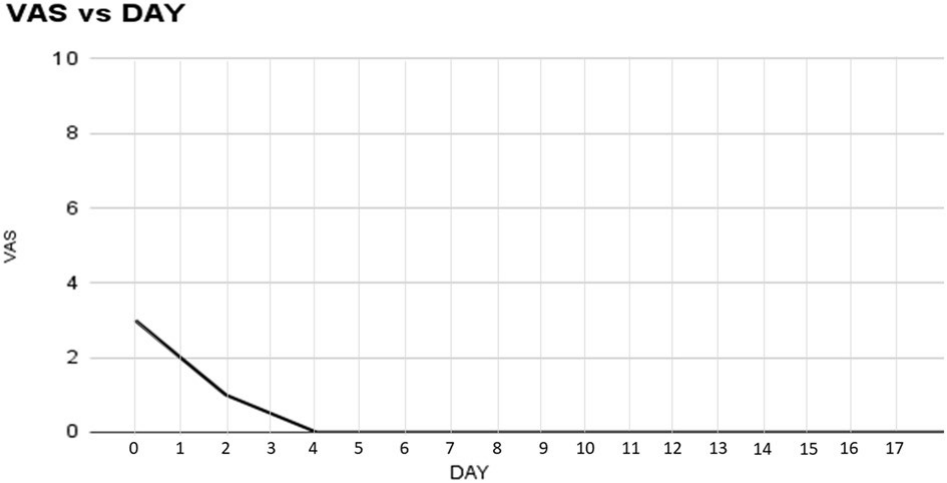
Postoperative mean VAS score assessment after laparoscopic hernia defect obliteration with ProFlor. Within 4th postoperative day all patients were pain-free.

**Figure 9:**
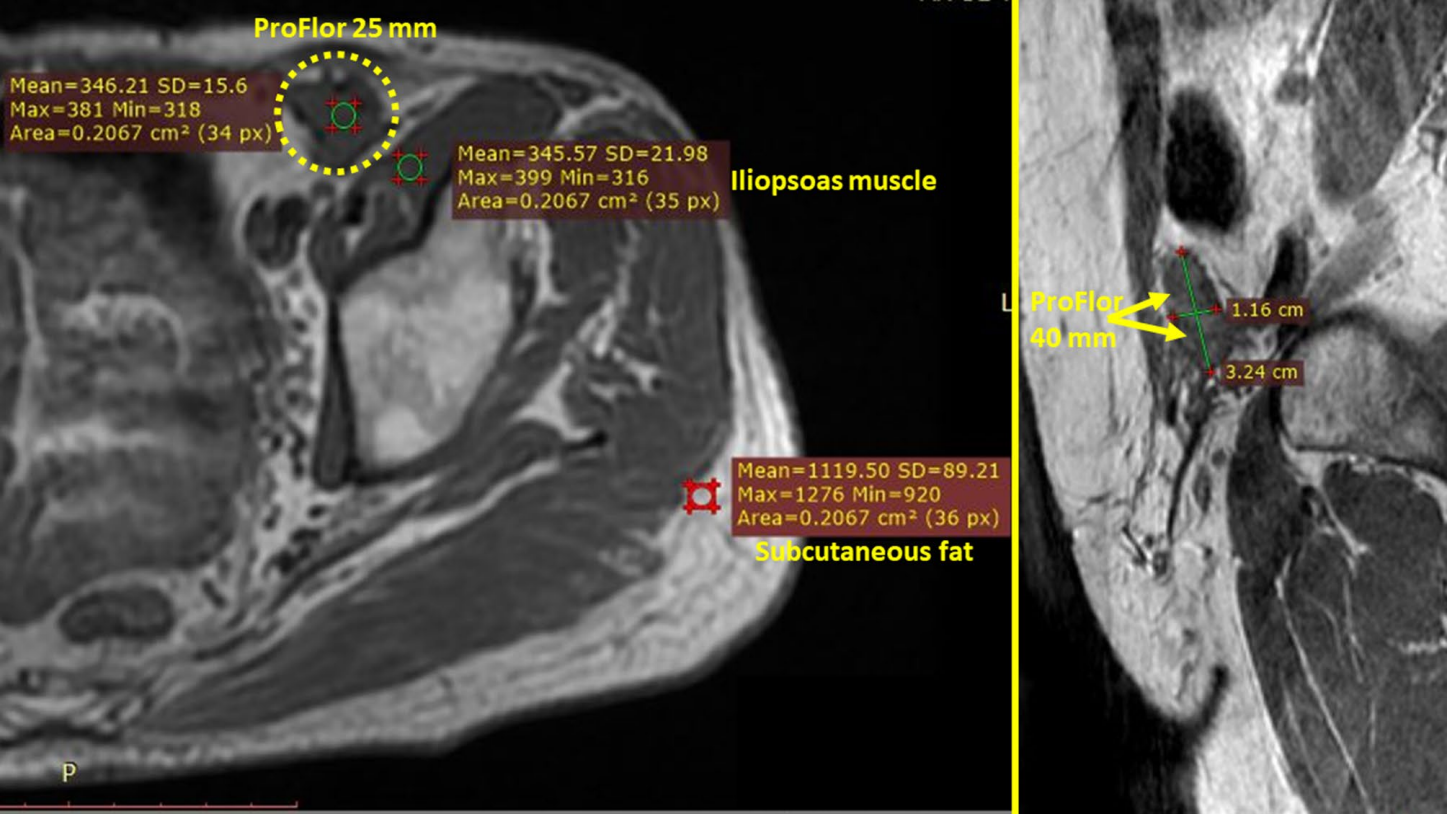
(**A**) Axial T1 SE MR image of the pelvis. (**A**) Patient with left inguinal hernia defect repaired with 25 mm ProFlor 9 months after implantation. 3D scaffold (yellow circle) occupies former hernia defect in the groin and appears wholly integrated into the inguinal backwall. 3D structure appears fully incorporated by newly ingrown tissue. The mean signal intensity (SI) of the newly developed tissue within ProFlor is nearly identical to the SI of the neighboring left iliopsoas muscle. Subcutaneous fat tissue of the lateral abdominal wall shows significantly higher SI. (**B**) Detail of sagittal T1 SE MR image of the pelvis showing a 40 mm ProFlor 12 months after implantation. 3D hernia device (yellow arrows) fully obliterates the defect. Transverse thickness of 3D scaffold, originally 15 mm, under steady pressure of visceral content, is reduced to approx. 11 mm, which is comparable to the thickness of the muscular barrier of the groin. Diameter of ProFlor, originally 40 mm, being squeezed within the former hernia defect now measures 32 mm.

The described 3D scaffold was employed to obliterate inguinal hernia defects fixation free in all 71 patients of the cohort. Despite no preoperative selection being made, from the preliminary US/CT imaging all patients met the inclusion criteria showing defect dimensions < 37 mm. This allowed the use of both 3D scaffold types with the 25- and 40-mm sized 3D core. Concerning hernia types, 33 patients of the cohort had unilateral recurrent hernias, two of whom with concomitant femoral hernia (forgotten hernia). 34 individuals presented with bilateral primary hernias, 6 others with double ipsilateral hernia in one groin and two additional patients with a double ipsilateral hernia in both groins (Figs. 6 A,B, 7). Three patients with bilateral hernias also had a concomitant femoral hernia. A total of 122 hernia defects were obliterated with 119 scaffolds since three double ipsilateral defects could be obliterated with just one 40 mm 3D scaffold that simply displaced laterally the divisor septum between the defects. With regard to location, 79 defects were in the lateral fossa, 24 in the medial fossa, 8 in the supravesical fossa, 6 were combined involving two neighboring fossae and an additional five in the femoral ring. Concerning the types of 3D scaffold used, 53 units were 25 mm in size and 66 units sized 40 mm in diameter. All details of the intraoperative findings, as well as the scaffolds utilized, are highlighted in Table 2. The mean duration of the operative procedure in unilateral (all recurrent) hernias was around 55 min (range 45–65 min.), in bilateral hernia, with single defect each side, the procedure lasted approximately 65 min (range 55–75 min) while in the cases with additional ipsilateral hernia mean time length was 75 min (range 65–85 min.). The time needed for obliterating the three reported femoral hernias was approximately ten additional minutes for each scaffold deployment. Overall, no intraoperative adverse events occurred. All patients were discharged the day after the operation. In the postoperative period, only 3 seromas (4.2%) were reported, and these were successfully managed conservatively. No hematomas, sepsis or other common adverse events occurred. During long-term follow-up, US scans showed no dislocation or migration of the 3D scaffold, which remained firmly in place fully obliterating the defect. The return to daily activities was nearly immediate, starting already from 43th to 5th postoperative day. No sense of foreign body, discomfort, or chronic pain was reported among the operated patients, even long term. In a follow up, from 36 to 6 months, no recurrence occurred in any patients of the cohort. All data of the postoperative outcomes are detailed in Table 3. Postoperative pain, assessed with the VAS scale, showed very low-pain scores starting from the first post-operative day. Regardless of unilateral recurrent or bilateral inguinal hernia repair with the 3D dynamic responsive scaffold, four days postop. all patients were pain-free (Fig. 8). Between 6 and 12 months postop. all individuals underwent MRI control of the lower pelvis to document the permanence in situ of the 3D scaffold obliterating the defect, as well as the quality of the newly developed tissue into the 3D scaffold by means of comparative signal intensity (Fig. 9A,B).

**Table 2:** Hernia types, locations and scaffolds used.

Hernia types		Total
Patients		71
Recurrent inguinal		33
Primary bilateral		
Single each side	68	84
Multiple ipsilateral*	16	
Femoral		5
Obliterated defects		122

Hernia location		Total
Lateral fossa		79
Medial fossa		24
Supravesical fossa		8
Combined		6
Femoral ring		5
Hernia defect site		
64 right/58 left		

ProFlor scaffolds used**		
25 mm		53
40 mm		66
Total		119

*Six patients had a double ipsilateral hernia in one groin and two other patients presented with double bilateral ipsilateral hernias.

**Three double ipsilateral hernias were repaired with a single unit of 40 mm ProFlor by side dislocation of the septal arrangement dividing the two defects (septum inguinalis).

**Table 3:** Results of laparoscopic ProFlor technique in examined patient cohort.

ProFlor laparoscopic: results		
Mean duration of surgical procedure		Minutes
Recurrent inguinal		50 (range 45–65)
Primary bilateral		
Single each side	54	65 (range 55–75)
Multiple ipsilateral	12	75 (range 65–85)
Femoral*		Additional 10 min

Postoperative complications—follow up 36–6 months	Total	%
Seroma	3	4.2%
Infection	0	0%
Discomfort	0	0%
Chronic pain	0	0%
Recurrence	0	0%
Total	3	4.27
Return to normal activity (days)	4 (mean)	Range 3–5

*All three femoral defects were concomitant hernias. One was detected during recurrent inguinal hernia repair and the remaining two during bilateral repair procedures.

## Discussion

Laparoscopic inguinal hernia repair is gaining increasing popularity among surgeons. However, this surgical procedure suffers from the same limitations as the open approach. As in conventional open prosthetic herniorrhaphy, uncontrolled foreign body response and mesh fixation are the main concerns^28–34^. The laparoscopic use of very broad meshes, often made of heavy-weight texture, logically intensifies the foreign body response induced by the biocompatible material causing an uncontrolled fibrotic apposition upon and around the flat mesh^35,36^. Postoperatively, the effects of excessive fibrotic ingrowth can lead to discomfort during movements and/or increased risks of groin nerve entrapment resulting in chronic pain. Concerning fixation, apart from expensive, short acting fibrin glue or the seemingly toxic cyanoacrylate adhesives^37,38^, in laparoscopic herniorrhaphy (both TEP and TAPP) mesh fastening is commonly carried out with metallic or resorbable tacks. This implies an evident negative impact on the abdominal wall physiology, as the screwed tacks, sharply piercing the delicate muscular surround, logically hinder groin kinetics^39,40^. Postoperatively, the pressure of the abdominal content puts the mesh under stress. If the hernia opening is larger than 2 cm, visceral pressure pushes the implant towards this weak point with possible detachment of the fixation tools. This may lead to mobilization and invagination of the mesh into the patent defect and is considered the main cause of recurrence in laparoscopic inguinal hernia repair^6^. If detached, tacks may tear the musculature causing bleeding and increased postoperative pain. Unfastened tacks falling in the preperitoneal space can migrate into the abdominal cavity leading sometimes to serious adverse events such as intestinal perforation^41,42^. Nevertheless, by critically analyzing the literature, it appears evident that, despite its significance, the issue of defect patency does not seem to be taken into adequate consideration. Years of scientific evidence have confirmed that the larger the defect the more recurrences occur, explaining why patency of the hernial opening continues to represent an unresolved and disappointingly undebated dilemma. To resolve the problem, some surgeons envisage that choosing larger and harder meshes plus increasing tack fixation could be the solution. However, this empirical solution does not appear to have met the needs. Therefore, it would seem evident that hernia defect patency is a crucial issue still waiting for a solution, as in clinical and experimental literature there are no reports that deal with the basic question: how can permanent obliteration of the hernia defect in laparoscopic groin hernia repair be achieved? In recent years, a different line of thought concerning the treatment of inguinal hernia has emerged based on updated evidence in physiopathology and the degenerative genesis of inguinal protrusions^43–50^. Following these new findings, a newly designed 3D device with dynamic responsivity has been developed for inguinal hernia repair. Made of the same polypropylene material as conventional meshes and owing an intrinsic centrifugal expansion, this inguinal hernia device, named ProFlor, is intended to be inserted fixation free into the defect thus achieving permanent obliteration. Recent literature has demonstrated that the dynamic compliance of this 3D hernia scaffold allows for a totally different biological response achieving development of newly formed connective tissue, vessels, nerves and muscle bundles, to full maturation^20–23^. The pathogenetically and physiologically coherent therapy model embodied by this 3D hernia device is diametrically opposed to the concept of reinforcing the groin with fibrotic mesh incorporation, which has been widespread for more than half a century. Conversely, the regenerative features of ProFlor mean that this 3D hernia device cannot be categorized as a conventional hernia prosthetics but rather as a regenerative scaffold. The above-depicted characteristics, undoubtedly uncommon for a hernia device, appear to demonstrate its effectiveness in the results achieved during the clinical study here presented. It should also be highlighted that on observing the size of the 3D scaffold, especially the 40 mm sized type used for laparoscopic hernia repair, it may be hard to believe that it can be introduced through a trocar channel into the abdominal cavity. Nevertheless, both clinical practice and specific physical characteristics of this hernia device make it possible to accomplish this crucial procedural step. As the 3D dynamic responsive scaffold used for laparoscopy is easily compressible, it can be enfolded by rolling the flat portion (Fig. 3A). With this configuration, it is possible to deliver both the 25 mm and the 40 mm types through a 12 mm channel by means of forceps (Fig. 3B–D). The laparoscopic procedure with this 3D hernia device is shorter and safer as, unlike the conventional laparoscopic approach, there is no need for a wide dissection of the inguinal backwall to deploy the meshes, usually 15 × 10 cm, to reduce the risks of invagination and/or recurrence^51^. On the contrary, achieving permanent defect obliteration renders wide dissection and deployment of large meshes superfluous, thus eliminating risks of injuries and reducing time needed. The 8 × 10 cm large, oval shaped, preperitoneal connected flat part of the 3D scaffold amply suffices for complete prophylactic coverage of the posterior inguinal area (Fig. 6), which surface ranges between 4.7 and 5.5 cm in length and 3–6.5 cm in height^52^. In addition, the low weight and very large porous texture of ProFlor’s flat portion, are far away from the mass and thickness of the meshes usually used for laparoscopic hernia repair also, like in double ipsilateral hernia may occur, in case of placement of two 3D devices in same groin.

At first glance, ProFlor’s procedural concept might resemble the plug-and-patch repair technique. Nevertheless, there are significant dissimilarities between these two models of treatment. Firstly, conventional plugs are static, not dynamic and, to avoid migration, must be fixated to the hernia opening. Further, plugs are inserted into the hernia opening mimicking defect obliteration, which, however results ineffective since within a few weeks the plug becomes incorporated by fibrotic fibers and shrinks up to 70%. These adverse phenomena do not occur in ProFlor that, positioned fixation free to permanently obliterate the defect, moves in tune with the inguinal backwall. Lastly, ProFlor does not shrink because its intrinsic dynamic responsivity induces a unique biologic response with development of newly formed vessels, nerves and muscle fibers characterizing this 3D device as a regenerative scaffold^20–24^. These proprietary features of ProFlor embody a substantial difference to patch and plug technique.

Analyses of the intra and postop. Outcomes show that no adverse events of note occurred in the described patient cohort. Reduced intraoperative trauma allowed all patients to be discharged within 24 h with no or negligible pain. Discomfort or pain intensity was sufficiently low for all individuals to return to the daily activity within 5 days. Except for three seromas, no major or minor complications, no chronic pain or recurrence postoperatively were reported. These positive results may be the consequence of a series of factors. Among these, the inherent dynamic behavior of the 3D scaffold is crucial, as it allows centrifugal expansion into the defect assuring permanent obliteration of the hernial opening without need for fixation. No fixation and dynamic compliance to groin movements are essential for a significant reduction of postoperative pain and avoid risk of developing chronic pain. US and MR imaging carried out during follow up demonstrated the permanence in situ of all dynamic scaffolds also in the long term. By fully and permanently occupying the hernia defect, the 3D hernia device impedes visceral re-protrusion through the inguinal barrier; hence no reported recurrences. Moreover, within a few months, the regenerative feature of the 3D scaffold leads to complete re-establishment of the inguinal barrier formerly wasted by hernia disease. The dynamically induced probiotic response of ProFlor in humans is clearly documented by the assessment of the signal intensity in MR imaging^53^. This, as seen in Fig. 9A,B, allows characterization of the enhanced quality of ingrown tissue in the 3D scaffold.

There are, however, some limitations which should be addressed in future studies. Firstly, even if statistically significant, the patient cohort is limited. This was mainly due to the negative impact of the recent COVID pandemic on elective surgery schedules. Furthermore, the study design also fully considered the recommendation of the EHS guidelines that strongly suggest laparoscopic inguinal hernia repair for recurrent hernias after open approach and for the treatment of bilateral inguinal hernias^54,55^. Therefore, patients with unilateral inguinal hernia were excluded from the study: these patients underwent open anterior hernia repair with the 3D scaffold in local anesthesia in the outpatient care system. Also, defects wider than 37 mm were excluded from the investigation. Whilst no preoperative selection was carried out, none of the preliminary US or CT assessed dimensions of the hernia defects was wider than 35 mm in the described cohort of patients. It must also be stated that more than 90% of hernia defects overall encountered in our clinical practice are less than 35 mm. For future cases of defects greater than 37 mm the planned solution involves delivering two 3D hernia devices connected together as previously documented in open ProFlor technique through a 12 mm channel^24^. Lastly, we are well aware that to be fully validated these results should be compared with those obtained with other laparoscopic techniques for inguinal hernia repair. However, the present report merely represents a preliminary study, which outcomes serve as a basic dataset for a comparative study with the TAPP technique carried out with conventional flat meshes, which is still ongoing.

## Conclusion

The described laparoscopic technique and the clinical results achieved with the 3D dynamic responsive and regenerative hernia scaffold seems to embody an innovative concept for the cure of inguinal hernias. Dynamic responsivity, fixation free deployment, permanent defect obliteration and regenerative behavior, are totally new features developed to overcome unphysiological and pathogenetically discrepant concepts of a therapy that was established more than half a century ago. In accordance with current international guidelines^56,57^, TAPP hernia repair technique with the 3D hernia scaffold ProFlor likely represents a concrete and promising alternative for decisively improving the laparoscopic treatment results of this widespread disease.

## Data Availability

The datasets used and analyzed during the study are available from the corresponding author upon reasonable request.
